# A 2021 Update on the Use of Liraglutide in the Modern Treatment of ‘Diabesity’: A Narrative Review

**DOI:** 10.3390/medicina57070669

**Published:** 2021-06-29

**Authors:** Mariana Cornelia Tilinca, Robert Aurelian Tiuca, Alexandru Burlacu, Andreea Varga

**Affiliations:** 1 Discipline of Internal Medicine, Department ME2, Faculty of Medicine, “George Emil Palade” University of Medicine, Pharmacy, Science and Technology, 540142 Targu Mures, Romania; mariana.tilinca@umfst.ro; 2 Compartment of Diabetology, Emergency Clinical County Hospital of Targu Mures, 540136 Targu Mures, Romania; 3 Clinic of Endocrinology, Mures County Clinical Hospital, 540072 Targu Mures, Romania; 4 Faculty of Medicine, ‘Grigore T. Popa’ University of Medicine and Pharmacy, 700115 Iasi, Romania; 5 Department of Interventional Cardiology, Cardiovascular Diseases Institute, 700503 Iasi, Romania; 6 Department ME2, Faculty of Medicine, “George Emil Palade” University of Medicine, Pharmacy, Science and Technology, 540136 Targu Mures, Romania; andreea.varga@umfst.ro; 7 Department of Cardiology II, Emergency Clinical County Hospital of Targu Mures, 540136 Targu Mures, Romania

**Keywords:** type 2 diabetes, obesity, GLP-1 RAs, liraglutide, diabesity

## Abstract

Obesity and type 2 diabetes mellitus have become a significant public health problem in the past decades. Their prevalence is increasing worldwide each year, greatly impacting the economic and personal aspects, mainly because they frequently coexist, where the term “diabesity” may be used. The drug class of glucagon-like peptide 1 receptor agonists (GLP-1 RAs) is one of the most modern therapy options in managing these metabolic disorders. This review focuses on the effects of liraglutide, a long-acting GLP-1 RA, in diabesity and non-diabetic excess weight. This drug class improves glycemic control by enhancing insulin secretion from the beta-pancreatic cells and inhibiting glucagon release. Furthermore, other effects include slowing gastric emptying, increasing postprandial satiety, and reducing the appetite and food consumption by influencing the central nervous system, with weight reduction effects. It also reduces cardiovascular events and has positive effects on blood pressure and lipid profile. A lower-dose liraglutide (1.2 or 1.8 mg/day) is used in patients with diabetes, while the higher dose (3.0 mg/day) is approved as an anti-obesity drug. In this review, we have summarized the role of liraglutide in clinical practice, highlighting its safety and efficacy as a glucose-lowering agent and a weight-reduction drug in patients with and without diabetes.

## 1. Introduction

Obesity and type 2 diabetes mellitus (T2DM) are significant public health issues, with both conditions presenting a yearly increasing prevalence worldwide, reaching pandemic proportions that imply economic and personal costs [1]. The use of glucagon-like peptide 1 receptor agonists (GLP-1 RAs) is one of the most modern therapy options in managing these metabolic disorders. The latest data by the World Health Organization (WHO) shows that the prevalence of obesity has tripled since 1975, with over 650 million people worldwide being obese in 2016 [2]. Obesity is typically defined by a body mass index (BMI) of ≥30 kg/m^2^, although a lower cutoff point of ≥27.5 kg/m^2^ is used in Asian populations [3]. The International Diabetes Federation (IDF) estimated in 2019 that 1 in 11 adults aged 20–79 years (463 million) have diabetes mellitus, with a projected number of more than 700 million cases by 2045 [4]. T2DM accounts for at least 90% of diabetes mellitus cases [5,6].

In 1973, the term “diabesity” was first used by Sims et al. to describe the pathophysiological connection between excess weight and T2DM [7]. Diabesity management is challenging because several types of glucose-lowering medications that are used in T2DM, such as sulfonylureas, meglitinides, thiazolidinediones, and insulin (including insulin analogs), could cause weight gain [8]. Increasing physical activity and following a calorie restriction diet are the main pillars for weight reduction [9]. Weight loss of at least 5% of baseline level in obese patients is clinically significant and improves several obesity-related cardio-metabolical complications, such as arterial hypertension, dyslipidemia, and impaired glucose metabolism (including prediabetes and T2DM) [10,11,12]. Pharmacological treatment is generally recommended in patients who did not present significant improvement after lifestyle modifications and BMI ≥ 30 kg/m^2^ or ≥27 kg/m^2^, with excess weight-associated comorbidities such as diabesity [1,13,14]. Currently approved anti-obesity drugs are illustrated in Table 1.

GLP-1 RAs and sodium-glucose co-transporter type 2 (SGLT-2) inhibitors are usually the class of drugs preferred in managing diabesity in association with metformin due to their association with weight loss [13,15]. In the present article, we aim to review and highlight the existing literature on the effects of liraglutide in managing diabesity and excess-weight individuals without diabetes, emphasizing its role as a lower-glucose therapy with benefits on cardiovascular outcomes, weight reduction, and quality of life.

## 2. GLP-1 Receptor Agonists

### 2.1. Physiology and Mechanism of Action of GLP-1

**Figure 1:**
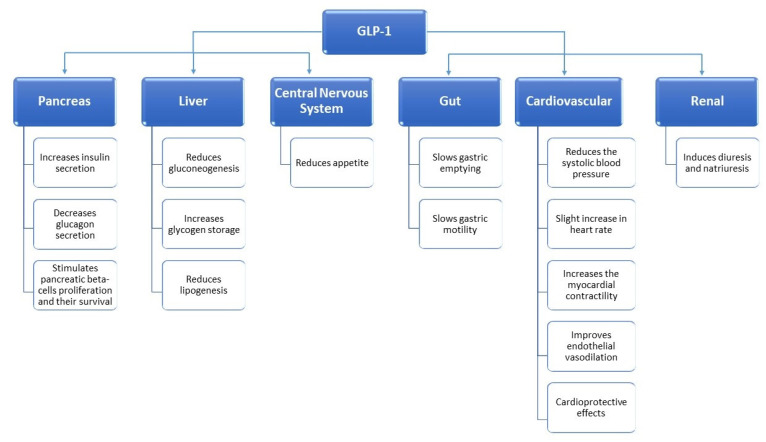
The main biological effects of Glucagon-like peptide 1(GLP-1).

GLP-1 is an endogenous incretin hormone secreted by small intestinal L cells in the distal ileum and proximal colon after ingestion of glucose and other carbohydrates, having low concentrations during fasting. GLP-1 receptors belong to the G-protein coupled receptor family and are mainly expressed in the pancreas, central nervous system, and gastrointestinal tract, also being found in the heart, kidneys, blood vessels, and peripheral nervous system [16]. Once GLP-1 binds to its receptor, the pancreatic beta-cells increase insulin production in a glucose-dependent manner due to enhanced intracellular levels of cyclic adenosine monophosphate (cAMP) [17]. It also promotes the survival and proliferation of pancreatic beta-cells [18,19]. Furthermore, GLP-1 inhibits glucagon secretion by pancreatic alfa-cells, reducing hepatic glucose production [20]. Other biological effects of endogenous GLP-1 include slowing gastric emptying, increasing postprandial satiety, and reducing appetite and food consumption by interfering in the central nervous system [21,22]. GLP-1 influences the neurons of the paraventricular and arcuate nucleus of the hypothalamus, attenuating orexigenic signals and reducing appetite [23,24]. GLP-1 has been shown to have cardioprotective effects associated with a reduction in systolic blood pressure, beneficial effects in ischemic cardiac injury or heart failure, and improvements in dyslipidemia [25,26,27]. Additionally, from a renal point of view, it induces diuresis and natriuresis [28]. The main biological effects of GLP-1 are summarized in Figure 1 [29].

The biological effects are limited by the short half-life of fewer than 2 min due to the rapid degradation of the endogenic GLP-1 by dipeptidyl peptidase IV (DPP4) [30,31,32].

### 2.2. Development, Pharmacokinetics, and Availability of GLP-1 Receptor Agonists

Given the beneficial effects of GLP-1 on glucose metabolism, its weight-loss potential, and the cardioprotective properties, there was an immense interest in developing pharmacological compounds that could mimic the action of GLP-1 and be resistant to the degradation of DPP-4 in order to be used in T2DM management. The first GLP-1 receptor agonist approved for T2DM treatment was the synthetic exenatide-4, eventually named exenatide, which was developed using the venom of the Gila Monster lizard.

Based on their pharmacological kinetics, GLP-1 receptor agonists are classified as short- or long-acting agents. Short-acting compounds are characterized by periods during which patients are exposed to circulating drug concentration that lasts for a few hours, followed by periods of GLP-1 inactivity. On the contrary, long-acting compounds produce a long-lasting circulating drug concentration with minor drug fluctuations. Given their pharmacokinetics, long-acting GLP-1 agonists have a greater capacity to lower the fasting plasma glucose than short-acting agents. The primary mechanism of glucose-lowering for short-acting GLP-1 Ras is defined by the slowed gastric emptying, while long-acting GLP-1 RAs lower the glucose by increasing insulin production and inhibiting glucagon. Short-acting GLP-1 RAs are exenatide (Byetta) and lixisenatide (Lyxumia, Adlyxin). Long-acting GLP-RAs are liraglutide (Victoza, Saxenda), once-weekly exenatide (Bydureon), dulaglutide (Trulicity), albiglutide (Eperzan, Tanzeum), semaglutide (Ozempic), and oral semaglutide (Rybelsus). GLP-1 RAs approved for use in T2DM treatment are summarized in Table 2 [33,34,35].

## 3. Liraglutide in Diabesity Management

Liraglutide’s (Victoza) safety and efficacy were assessed in the Liraglutide Effect and Action in Diabetes (LEAD) program, including 6 phase III randomized clinical trials. Liraglutide was studied in this program alone or in combination therapy with other antidiabetic drugs.

### 3.1. Liraglutide in Blood Glucose Control

LEAD-1 included 1041 subjects with T2DM and compared the combination of liraglutide 0.6, 1.2, and 1.8 mg/day, or rosiglitazone 4 mg/day or placebo, with glimepiride for 26 weeks. When added to glimepiride, liraglutide (1.2 or 1.8 mg/day) had a greater reduction in glycated hemoglobin (HbA1c) when compared to placebo (*p* ≤ 0.0001) or to rosiglitazone (*p* ≤ 0.0001). Postprandial glucose was significantly improved with liraglutide 1.2 or 1.8 mg compared to placebo (*p* ≤ 0.0001) or to rosiglitazone (*p* ≤ 0.05) [36].

In the LEAD-2 trial, which included 1091 subjects, liraglutide was compared with placebo and glimepiride 4 mg/day. All treatments were administered in combination with metformin 2000 mg/day. Liraglutide-treated subjects were superior to placebo and non-inferior to glimepiride when referring to glycemic control [37]. Liraglutide was also compared with insulin glargine and placebo during the LEAD-5 trial, including 581 subjects with T2DM with prior monotherapy and combination therapy. Liraglutide 1.8 mg/day had a greater reduction in HbA1c compared to insulin glargine at 26 weeks (−1.33% vs. −1.09%, *p* = 0.0015), with similar reductions in FBG and postprandial glucose [38]. A comparison between liraglutide 1.8 mg and twice-daily 10 μg exenatide was conducted in the LEAD-6 study in adults with uncontrolled T2DM on maximum doses of metformin and sulfonylurea. Liraglutide reduced mean HbA1c significantly more than exenatide (−1.12% vs. −0.79%; estimated treatment difference (ETD) −0.33%, *p* ≤ 0.0001). FBG was reduced more in liraglutide-treated subjects than exenatide (−1.61 mmol/L vs. −0.60 mmol/L; ETD −1.01 mmol/L, *p* ≤ 0.0001). More patients with liraglutide achieved HbA1c < 7% compared with exenatide (54% vs. 43%; *p* = 0.0015) [39].

### 3.2. Liraglutide in Body Weight Management

Liraglutide can activate GLP-1 receptors found in hypothalamic regions that control feeding, such as the hypothalamic paraventricular and arcuate nucleus. For example, in the arcuate nucleus, liraglutide stimulates the appetite-inhibiting pro-opiomelanocortin neurons and inhibits the orexigenic neuropeptide-Y and Agouti-related peptide neurons, resulting in reduced food intake and increased satiety [40].

Liraglutide has proven its weight loss benefits in several clinical trials. In the LEAD-2 trial, body weight loss in a dose-dependent manner was noted in the liraglutide group (up to 2.8 kg) compared to the glimepiride group, that had associated weight gain [37]. In the LEAD-5 trial, liraglutide produced significant weight loss vs. placebo (treatment difference −1.39 kg, *p* = 0.0001) and vs. insulin glargine (treatment difference −3.43 kg, *p* = 0.0001) [38]. In the LEAD-6 trial, liraglutide 1.8 mg/day and exenatide 10 μg twice-daily produced similar weight loss outcomes (−3.24 kg vs. −2.87, *p* = 0.22) [39].

The Satiety and Clinical Adiposity-Liraglutide Evidence (SCALE) clinical trial conducted at 126 sites in 9 countries investigated the safety and efficacy of liraglutide as a weight-reducing drug in excess-weight individuals with T2DM, in association with a 500 kcal/day deficit and increased physical activity. After 56 weeks, in subjects with T2DM, 3.0 mg of daily liraglutide was associated with the most weight loss when compared to liraglutide 1.8 mg/day and placebo (6.4 kg vs. 5.0 kg vs. 2.2 kg; ETD for liraglutide 3.0 vs. placebo—4.00%; ETD for liraglutide 1.8 vs. placebo −2.71%; *p* for both liraglutide ≤ 0.001) [41]. Weight loss of 5–10% of body weight is associated with improved glycemic control and improved outcome of comorbidities in T2DM. Weight loss of at least 5% occurred in 54.3% with liraglutide 3.0 mg, 40.4% with liraglutide 1.8 mg, and 21.4% with placebo (*p* for both liraglutide ≤ 0.001). Furthermore, a reduction of at least 10% in weight loss was noted in 25.2% of cases with liraglutide 3.0 mg, 15.9% with liraglutide 1.8 mg, and 6.7% with placebo (ETD between liraglutide 3.0 mg vs. placebo, *p* ≤ 0.001; for liraglutide 1.8 mg vs. placebo, *p* = 0.006) [41].

The SCALE Obesity and Prediabetes double-blinded trial investigated the possible benefits of liraglutide 3.0 mg/day for 56 weeks in 3731 individuals without T2DM with a BMI higher than 30 or 27 kg/m^2^ when dyslipidemia or hypertension are present. At week 56, liraglutide-treated subjects achieved a weight loss of 8.4 ± 7.3 kg vs. placebo group with a loss of 2.8 ± 6.5 kg (ETD –5.6 kg, *p* ≤ 0.001). Furthermore, 63.2% of subjects in the liraglutide group had a weight loss of at least 5% from baseline vs. 27.1% of subjects in the placebo group (*p* ≤ 0.001). A weight loss of at least 10% was encountered in 33.1% in the liraglutide group vs. 10.6% in the placebo group (*p* ≤ 0.001) [42].

Based on the SCALE clinical trial results, liraglutide alongside lifestyle changes has beneficial effects not only in T2DM but also in patients without diabetes by promoting weight loss and improving metabolic control, meaning it can prevent the development of T2DM in this category of patients. Therefore, given its positive outcomes on weight loss, liraglutide 3.0 mg was approved by both the Food and Drugs Administration (FDA) (2014) and European Medicines Agency (EMA) (2015) as an anti-obesity drug in patients without diabetes sold under the name of Saxenda, making it the first GLP-1 RA approved for use in chronic weight management. Recently, the FDA approved the use of Semaglutide 2.4 mg/once weekly (June 2021) as a chronic weight management drug in adults with obesity or overweight with at least one weight-related condition after proving its weight benefits in several clinical trials [43,44,45,46].

Liraglutide also shows very promising perspectives in the pharmacological management of obesity in individuals under 18 years of age. In 2020, a randomized double-blinded trial, which investigated the use of liraglutide 3.0 mg as a weight reduction option in obese adolescents, published its results. Liraglutide was found to be superior to placebo plus lifestyle therapy in reducing the BMI standard deviation score at week 56 (ETD –0.22%; 95% confidence interval (CI) −0.37 to −0.08; *p* = 0.002) [47]. A randomized, placebo-controlled trial aimed to investigate the maintenance of weight loss achieved through caloric deficit followed by a 1-year treatment with liraglutide, physical activity, or both, compared with placebo in individuals with obesity. In the recently published results, liraglutide 3.0 mg/day when combined with physical activity greatly improved weight loss maintenance compared with liraglutide alone or physical exercise alone (−9.5 kg vs. −6.8 kg vs.−4.1 kg) [48]. These results strengthen the weight loss benefits of liraglutide and emphasize the critical role of physical activity in chronic weight management.

### 3.3. Liraglutide and Cardiovascular Outcomes

In the LEAD-5 clinical trial, liraglutide had a beneficial effect on systolic blood pressure vs. insulin glargine (−4.5 mmHg difference, *p* = 0.0001), although not when compared to placebo (*p* = 0.0791) [38]. The cardiovascular outcomes of liraglutide were investigated in the LEADER trial (Liraglutide Effect and Action in Diabetes: Evaluation of cardiovascular outcome Results): 9340 subjects with T2DM and high cardiovascular risk were randomized and followed for a median of 3.8 years. The LEADER trial noted that primary outcomes (cardiovascular death, nonfatal myocardial infarction, or nonfatal stroke) occurred less in the liraglutide group (608 out of 4668 (13.0%)) vs. placebo (694 out of 4672 (14.9%)) (hazard ratio (HR) 0.87; 95% CI 0.78–0.97; *p* = < 0.001 for non-inferiority; *p* = 0.01 for superiority). Furthermore, death from cardiovascular causes occurred less frequently in the liraglutide group vs. placebo (4.7% vs. 6.0%; *p* = 0.007). All-cause mortality was reduced in the liraglutide group vs. placebo (8.2% vs. 9.6%; *p* = 0.02). Nonfatal myocardial infarction and nonfatal stroke were less frequent in the liraglutide group than in the placebo group; however, the difference was not significant. Moreover, heart failure hospitalization rates were lower in the liraglutide group but without a significant difference vs. the placebo group [49]. Aiming to evaluate the effects of liraglutide on cardiovascular events and mortality based on patients’ heart failure history, T2DM participants in the LEADER trial were assigned through randomization to placebo or liraglutide (1.8 mg daily or a maximum tolerated dose up to 1.8 mg daily). The risk of heart failure hospitalization was not increased in the liraglutide group vs. placebo, regardless of heart failure history at baseline. Moreover, use of liraglutide when compared to placebo was associated with lower rates of major cardiovascular events, nephropathy, and mortality, and therefore, liraglutide should be considered appropriate for use in patients with diabetes with or without a history of heart failure (New York Heart Association functional class I to III) [50].

In a post hoc analysis of the LEADER trial 1, the cardiovascular effects of liraglutide vs. placebo in patients with or without preexisting metformin treatment were analyzed. It was noted that the incidence of primary outcomes (time from randomization to first occurrence of cardiovascular death, myocardial infarction, or stroke) was not significantly reduced in liraglutide users when compared to placebo among previous metformin users (HR adjusted 0.97; 95% CI: 0.85–1.10), but liraglutide did reduce the incidence among metformin non-users (HR adjusted 0.79; 95% CI: 0.64–0.97), highlighting a possible role that metformin could have in enhancing the cardiovascular benefits of GLP-1 RAs [51].

A systematic review and meta-analysis which included 8 studies published between 2009 and 2016 and a total of 14,608 patients with T2DM showed that patients who underwent liraglutide treatment had a lower risk compared to comparison groups of developing major cardiovascular events (MACE), acute myocardial infarction, and cardiovascular death, but not a decreased incidence of stroke. However, regarding MACE, a significant decreased incidence with liraglutide was noticed only in trials using placebo (relative risk (RR) = 0.89, 95% CI: 0.83–0.96, *p* = 0.004) and not in those using other comparators (RR = 0.58, 95% CI: 0.29–1.16, *p* = 0.122) [52].

A register-based study performed in Denmark and Sweden analyzed the risk of major cardiovascular events between patients with T2DM treated with liraglutide and dipeptidyl peptidase-4 (DPP-4) inhibitors. Liraglutide had a significantly lower risk of cardiovascular death compared with DPP-4 inhibitors (HR: 0.78, 95% CI: 0.68–0.91) and a significantly lower risk of death from any cause (HR: 0.83, 95% CI: 0.77–0.90). However, no significant differences were observed for risk of myocardial infarction (HR: 0.94, 95% CI: 0.84–1.06), stroke (HR: 0.88, 95% CI: 0.77–1.01), risk of heart failure (HR: 0.90, 95% CI: 0.80–1.03), or for other major cardiovascular event outcomes (HR: 0.95, 95% CI: 0.89–1.01) [53].

A post hoc analysis using data from 5908 participants with obesity in 5 SCALE randomized controlled trials (liraglutide vs. placebo or orlistat) revealed that liraglutide 3 mg was not associated with an increased risk of cardiovascular events (HR: 0.42; 95% CI: 0.17–1.08) [54].

A summary of the presented clinical trials and their primary outcomes is illustrated in Supplementary Table S1.

## 4. Liraglutide Side Effects

### 4.1. Gastrointestinal Side Effects and Concerns

Liraglutide side effects are shared with other GLP-1 RAs. The most common side effects reported with GLP-1 RAs treatment are gastrointestinal areal, nausea, vomiting, and diarrhea, being the most frequent signs and symptoms reported. Most commonly, these side effects occur after the initiation of the pharmacological treatment and after increasing the dose. Therefore, to minimize the impact of these adverse events, it is recommended that every GLP-1 RA be initiated at a lower dose, then slowly increase the dose through up-titration regimens.

The mild elevation in amylase and/or lipase, together with the abdominal discomfort, both situations commonly found in GLP-1 RA-treated individuals, led to the concerns of pancreatitis [55]. Liraglutide 1.8 mg showed a 28% increase in lipase levels and a 7% increase in amylase levels compared to placebo, while liraglutide 3.0 mg had a 30% increase in lipase levels and a 7% increase in amylase levels [56]. A meta-analysis from 2017 confirmed the safety of GLP-1 RAs for pancreatitis, reporting that the incidence of pancreatitis and pancreatic cancer was not significantly different between GLP-1 RAs and comparators (odds ratio (OR) 0.93; 95% CI 0.65–1.34; *p* = 0.71, and odds ratio 0.94; 95% CI 0.52–1.70; *p* = 0.84) [57].

Interestingly, a meta-analysis from 2018 documented the possibility of protective properties of incretin drugs against pancreatic cancer in T2DM patients who followed incretin-based antidiabetic treatment ≥ 104 weeks (OR 0.62; 95% CI 0.41–0.95) [58]. A recent meta-analysis based on cardiovascular outcomes trials found no significant risk of acute pancreatitis nor for any malignant neoplasms [59]. Nevertheless, it is recommended to avoid GLP-1 RAs in patients with pancreatic cancer or pancreatitis [60]. A recent meta-analysis pointed out a significant risk of cholelithiasis in liraglutide-treated patients (0.6–3.0 mg) [61].

### 4.2. Hypoglycemia

Liraglutide is associated with a low risk of hypoglycemia. GLP-1 RAs increase insulin secretion and inhibit the glucagon action in a glucose-dependent manner, making it less likely for hypoglycemia [62]. Nevertheless, the use of liraglutide in combination with insulin or sulfonylureas may increase the hypoglycemia risk [36,37,38].

### 4.3. Medullary Thyroid Cancer Concerns

There were concerns about a possible increased risk of developing medullary thyroid carcinoma (MTC) after an association was noted in rodent studies between long-acting GLP-1 RA (such as liraglutide) and thyroid parafollicular C-cell hyperplasia. However, in humans, these concerns have not been confirmed so far as no such association between liraglutide and MTC has been detected in any clinical trials to date. One explanation for the association between MTC and liraglutide seen in rodents and not in humans could be that rodents’ parafollicular C-cells have more GLP-1 receptors [63]. Nevertheless, GLP-1 RAs are not recommended in subjects at high risk of developing MTC, such as a known personal or family history of MTC and/or multiple endocrine neoplasia type 2A and 2B [33,60].

### 4.4. Kidney Function

Investigating the kidney function before initiating treatment with GLP-1 RAs is highly important, by measuring the creatinine levels and the estimated glomerular filtration rate (eGFR). Liraglutide is safe to use in moderate renal impairment (eGFR: 30–60 mL/min/1.73 m^2^), while in severe (eGFR: 15–30 mL/min/1.73 m^2^) and end-stage renal disease (eGFR: <15 mL/min/1.73 m^2^), it should be used with caution due to limited data [64]. There have been reports of acute kidney injury in chronic kidney disease individuals following treatment with GLP-1 RAs. The use of diuretics or renin-angiotensin-aldosterone system inhibitors in the presence of vomiting and established chronic kidney disease could potentially complicate the GLP-1 RAs treatment [60].

## 5. Conclusions

Diabetes mellitus and obesity are two metabolic disorders whose prevalence have significantly increased over the past decades and are often coexisting. Liraglutide is an efficient and safe option in diabesity treatment, proving in several studies its safety and efficacy as a glucose-lowering agent and a weight reduction drug in individuals with and without diabetes. Having relatively low contraindications and cardiovascular benefits, liraglutide is a modern option in fighting the diabesity pandemic.

## Figures and Tables

**Table 1:** Currently approved anti-obesity pharmacological treatment.

Drug Name, Posology	Mechanism of Action	FDA-Approved	EMA-Approved
orlistat, 60–120 mg 3 × 1 tablet daily	Pancreatic lipase inhibitor	YES (1999)	YES (1998)
phentermine/topiramate, 3.75/23 mg; 7.5/46 mg; 11.25/69 mg; 15/92 mg once daily	Sympathomimetic, appetite suppressant	YES (2012)	NO
naltrexone/bupropion, 32/360 mg 2 × 4 tablets daily	Opioid receptor antagonist/dopamine and noradrenaline reuptake inhibitor	YES (2012)	YES (2015)
liraglutide, 3.0 mg injection once daily	GLP-1 receptor agonist	YES (2014)	YES (2015)
semaglutide, 2.4 mg injection once weekly	GLP-1 receptor agonist	YES (2021)	NO

Abbreviations: mg, milligram; GLP-1: glucagon-like peptide 1; FDA, Food and Drug Administration; EMA, European Medicines Agency.

**Table 2:** GLP-1 RAs approved for T2DM treatment.

GLP-1 Agonist	Duration of Action	First Approval	Elimination Half-Life	Posology
Exenatide	Short-acting	2005 (USA) 2006 (Europe)	3.3–4.0 h	Subcutaneous injection, twice daily, before meals Dosage: 5 μg, after 1 month, increase to 10 μg
Lixisenatide	Short-acting	2013 (Europe) 2016 (USA)	2.6 h	Subcutaneous injection, once daily, before meals Dosage: 10 μg, after 15 days, increase to 20 μg
Liraglutide	Long-acting	2009 (Europe) 2010 (USA)	12.6–14.3 h	Subcutaneous injection, once daily Dosage: 0.6 mg, after 1 week, increase to 1.2 mg; it can be increased to 1.8 mg in case of poor glycemic control
Once-weekly Exenatide	Long-acting	2012	3.3–4.0 h	Subcutaneous injection, once weekly Dosage: 2 mg once weekly with or without meals
Dulaglutide	Long-acting	2014	4.7–5.5 days	Subcutaneous injection, once weekly Dosage: 0.75 mg once weekly; it can be increased to 1.5 mg in case of poor glycemic control
Albiglutide	Long-acting	2014	5.7–6.8 days	Subcutaneous injection, once weekly Dosage: 30 mg once weekly; it can be increased to 50 mg in case of poor glycemic control
Semaglutide	Long-acting	2017 (USA) 2018 (Europe)	5.7–6.7 days	Subcutaneous injection, once weekly Dosage: 0.25 mg once weekly, after 1 month, increase to 0.5 mg; it can be increased to 1 mg in case of poor glycemic control
Semaglutide	Long-acting	2019	5.7–6.7 days	Oral administration, once weekly, before meals Dosage: 3 mg, after 1 month, increase to 7 mg; it can be increased to 14 mg in case of poor glycemic control

GLP-1 RAs: glucagon-like peptide 1 receptor agonists; T2DM: type 2 diabetes mellitus.

## Supplementary Materials

The following are available online at https://www.mdpi.com/article/10.3390/medicina57070669/s1, Table S1: Main outcomes of LEAD, SCALE, and LEADER clinical trials.
