# Supplementary material for: A 2021 Update on the Use of Liraglutide in the Modern Treatment of ‘Diabesity’: A Narrative Review
Source: Medicina (Kaunas). 2021 Jun 29;57(7):669. doi: 10.3390/medicina57070669 (PMC8307742; doi:10.3390/medicina57070669)
Supplement: Supplementary file 1 [file medicina-57-00669-s001.zip › medicina-1284751-supplementary.pdf]

**Supplementary Table S1**. Main outcomes of LEAD, SCALE and LEADER clinical trials

| Clinical Trial | Aim | Main results |
|---|---|---|
| LEAD-1 | Compare the effects of combining liraglutide (0.6, 1.2, and 1.8 mg/day) or rosiglitazone 4 mg/day or placebo with glimepiride | The addition of liraglutide to glimepiride had a greater reduction in HbA1c vs placebo or rosiglitazone |
| LEAD-2 | Compare the efficacy and safety of liraglutide with placebo or glimepiride when given in combination with metformin | Liraglutide was superior to placebo and non-inferior to glimepiride in glucose control<br><br>Bodyweight loss was noted in the liraglutide group (up to 2.8 kg) |
| LEAD-5 | Compare the efficacy and safety of liraglutide with placebo and insulin glargine in combination with metformin and glimepiride | HbA1c had a greater reduction with liraglutide 1.8 mg/day vs insulin glargine<br><br>Liraglutide had a significant weight reduction vs placebo and vs insulin glargine<br><br>Liraglutide significantly reduced the systolic blood pressure vs insulin glargine |
| LEAD-6 | Compare the efficacy and safety of liraglutide with exenatide in combination with metformin, sulfonylurea, or both | HbA1c had a greater reduction with liraglutide 1.8 mg vs exenatide |
| SCALE diabetes clinical trial | Investigated the safety and efficacy of liraglutide in individuals with or without T2DM | Liraglutide 3.0 mg achieved the most weight loss vs liraglutide 1.8 mg vs placebo in T2DM |
| SCALE obesity and pre-diabetes trial | Investigated the benefits of liraglutide 3.0 mg in individuals without T2DM with BMI of at least 30 kg/m$^2$ or at least 27 kg/m$^2$ with dyslipidemia or hypertension | Liraglutide 3.0 mg was associated with significant weight loss vs placebo |
| LEADER | Investigate the cardiovascular outcomes of liraglutide | Liraglutide was associated with significantly less frequent primary outcome occurrence (first occurrence of death from cardiovascular causes, nonfatal myocardial infarction, or nonfatal stroke) vs placebo<br><br>Liraglutide had less deaths from any cardiovascular causes' vs placebo |
| Abbreviations | LEAD, Liraglutide Effect and Action in Diabetes; LEADER, Liraglutide Effect and Action in Diabetes: Evaluation of cardiovascular outcome Results; SCALE, The Satiety and Clinical Adiposity-Liraglutide Evidence; HbA1c, glycated hemoglobin; T2DM, type 2 diabetes mellitus; BMI, body mass index; mg, milligram; vs, versus | |
